# Author Correction: Somatic mutations in facial skin from countries of contrasting skin cancer risk

**DOI:** 10.1038/s41588-023-01508-6

**Published:** 2023-08-25

**Authors:** Charlotte King, Joanna C. Fowler, Irina Abnizova, Roshan K. Sood, Michael W. J. Hall, Ildikó Szeverényi, Muly Tham, Jingxiang Huang, Stephanie Ming Young, Benjamin A. Hall, E. Birgitte Lane, Philip H. Jones

**Affiliations:** 1 Wellcome Sanger Institute, Hinxton, UK; 2 Department of Oncology, University of Cambridge, Hutchinson Research Centre, Cambridge Biomedical Campus, Cambridge, UK; 3 Present Address: Skin Research Institute of Singapore and Institute of Medical Biology, Agency for Science, Technology and Research (A*STAR), Singapore, Singapore; 4 Department of Ophthalmology, National University Hospital, Singapore, Singapore; 5 Department of Medical Physics and Biomedical Engineering, University College London, London, UK; 6 Institute of Aquaculture and Environmental Safety, Georgikon Campus, Hungarian University of Agricultural and Life Sciences, Keszthely, Hungary

**Keywords:** Squamous cell carcinoma, Genetics research

Correction to: *Nature Genetics* 10.1038/s41588-023-01468-x. Published online 3 August 2022.

In the version of this article originally published, Ildikó Szeverényi (Skin Research Institute of Singapore and Institute of Medical Biology, Agency for Science, Technology and Research (A*STAR), Singapore, Singapore) was listed with an incorrect primary affiliation. The error has been corrected in the HTML and PDF versions of the article.

