# Supplementary material for: Somatic mutations in facial skin from countries of contrasting skin cancer risk
Source: Nat Genet. 2023 Aug 3;55(9):1440–7. doi: 10.1038/s41588-023-01468-x (PMC10484783; doi:10.1038/s41588-023-01468-x)
Supplement: Supplementary file 1 — Reporting Summary [file 41588_2023_1468_MOESM1_ESM.pdf]

nature portfolio

Corresponding author(s): Philip H. Jones

Last updated by author(s): Jun 20, 2023

Reporting Summary

Nature Portfolio wishes to improve the reproducibility of the work that we publish. This form provides structure for consistency and transparency in reporting. For further information on Nature Portfolio policies, see our Editorial Policies and the Editorial Policy Checklist.

Please do not complete any field with "not applicable" or n/a. Refer to the help text for what text to use if an item is not relevant to your study.
For final submission: please carefully check your responses for accuracy; you will not be able to make changes later.

Statistics

For all statistical analyses, confirm that the following items are present in the figure legend, table legend, main text, or Methods section.

n/a	Confirmed	
☐	☒	The exact sample size (*n*) for each experimental group/condition, given as a discrete number and unit of measurement
☒	☐	A statement on whether measurements were taken from distinct samples or whether the same sample was measured repeatedly
☐	☒	The statistical test(s) used AND whether they are one- or two-sided<br>*Only common tests should be described solely by name; describe more complex techniques in the Methods section.*
☒	☐	A description of all covariates tested
☐	☒	A description of any assumptions or corrections, such as tests of normality and adjustment for multiple comparisons
☐	☒	A full description of the statistical parameters including central tendency (e.g. means) or other basic estimates (e.g. regression coefficient) AND variation (e.g. standard deviation) or associated estimates of uncertainty (e.g. confidence intervals)
☐	☒	For null hypothesis testing, the test statistic (e.g. *F*, *t*, *r*) with confidence intervals, effect sizes, degrees of freedom and *P* value noted<br>*Give P values as exact values whenever suitable.*
☒	☐	For Bayesian analysis, information on the choice of priors and Markov chain Monte Carlo settings
☐	☒	For hierarchical and complex designs, identification of the appropriate level for tests and full reporting of outcomes
☐	☒	Estimates of effect sizes (e.g. Cohen's *d*, Pearson's *r*), indicating how they were calculated

Our web collection on statistics for biologists contains articles on many of the points above.

Software and code

Policy information about availability of computer code

Data collection	No software was used to collect data
Data analysis	BAM files were mapped to the GRCh37d5 reference using BWA-MEM (v0.7.17) and duplicate reads marked using Biobambam2 (https://gitlab.com/german.tischler/biobambam2, v2.0.86). Coverage was calculated using samtools (v1.14) and bedtools (v2.28.0). Mutations were called using deepSNV (https://github.com/gerstung-lab/deepSNV, v1.21.3) and annotated using VAGrENT (v3.3.3). All downstream analysis was conducted in R (v4.1.3) with data visualisation and statistical analysis conducted using R packages: Biostrings, car, deepSNV, dndscv (v0.0.1.0), GenomicRanges, ggrepel, ggpubr, igraph, MASS, plotrix, pvclust, Rsamtools, rstatix, seqinr and tidyverse. Mutational signature analysis was conducted using SigProfiler (v1.1.13). Germline variants were called using GATK (v4.3.0.0) best practices. Korean cSCC samples were obtained using SRA-toolkit (v2.10.9), assessed using FastQC (v0.11.2) and MultiQC (v1.13) and aligned to GRCh37d5 using BWA-MEM (v.0.7.17, https://github.com/lh3/bwa) and Biobambam2 (v.2.0.86, https://gitlab.com/german.tischler/biobambam2). Mutations were called using Caveman (v1.17.4) and Pindel (v3.3.0) and annotated using VAGrENT (v3.3.3). All modelling code is available at https://github.com/irinaabnizova/cell_competition_2D

For manuscripts utilizing custom algorithms or software that are central to the research but not yet described in published literature, software must be made available to editors and reviewers. We strongly encourage code deposition in a community repository (e.g. GitHub). See the Nature Portfolio guidelines for submitting code & software for further information.

Data

Policy information about availability of data

All manuscripts must include a data availability statement. This statement should provide the following information, where applicable:

- Accession codes, unique identifiers, or web links for publicly available datasets
- A description of any restrictions on data availability
- For clinical datasets or third party data, please ensure that the statement adheres to our policy

Sequencing data has been deposited at EGA and is accessible with the accession number EGAD00001009666, title: Somatic mutations in facial skin from countries of contrasting skin cancer risk.

Research involving human participants, their data, or biological material

Policy information about studies with human participants or human data. See also policy information about sex, gender (identity/presentation), and sexual orientation and race, ethnicity and racism.

Reporting on sex and gender	Male and female sex is reported and both are represented in the data. Males: n = 6, females: n = 5
Reporting on race, ethnicity, or other socially relevant groupings	Human donors were categorized by the country in which they live (Singapore and the UK). We then genotyped donors for relevant SNPs and showed donors were representative of the wider populations of each country.
Population characteristics	Donors were aged 28-79 years (Singapore mean = 62 years, UK mean = 68 years), with both males/females, smokers/non-smokers and indoor/outdoor workers from each country.
Recruitment	Participants were undergoing blepharoplasty or browplexy surgery in Singapore or the UK. This is performed for age-related loss of elasticity of the dermis. No other selection criteria were applied. Participants did not receive compensation and we are not aware of any recruitment bias that may impact the results.
Ethics oversight	Written informed consent was obtained in all cases. The study received ethical approval from the Nanyang Technological University Institutional Review Board (www.ntu.edu.sg/research/research-integrity-office/institutional-review-board/the-ntu-institutional-review-board-(irb), protocol number, NHG study 2016/00659-AMD000I). The UK component of the study received ethical approval under UK approved protocols (Research Ethics Committee references 15/EE/0152 NRES Committee East of England-Cambridge South and 15/EE/0218 NRES Committee East of England-Cambridge East).

Note that full information on the approval of the study protocol must also be provided in the manuscript.

Field-specific reporting

Please select the one below that is the best fit for your research. If you are not sure, read the appropriate sections before making your selection.

☒ Life sciences ☐ Behavioural & social sciences ☐ Ecological, evolutionary & environmental sciences

Life sciences study design

All studies must disclose on these points even when the disclosure is negative.

Sample size	Sample size was estimated from previous studies (PMID: 25999502, PMID: 33087317).
Data exclusions	No data were excluded from the study.
Replication	Multiple donors were sampled from each country, with a large effect size between countries. The scarcity of donated healthy human tissue makes the experiment difficult to replicate but the high consistency with previously published work gives us confidence on the reproducibility.
Randomization	Consecutive consenting eligible donors were recruited into the study without other selection. Allocation into experimental groups was based on the country in which the participant lives (our independent variable) and therefore randomization is not relevant to this study.
Blinding	Blinding is not relevant to data collection or data analysis in this study as DNA sequencing, mutation calling and downstream algorithms are not affected by investigator knowledge of a donor's country.

Reporting for specific materials, systems and methods

We require information from authors about some types of materials, experimental systems and methods used in many studies. Here, indicate whether each material, system or method listed is relevant to your study. If you are not sure if a list item applies to your research, read the appropriate section before selecting a response.

Materials & experimental systems

n/a	Involved in the study
☒	☐ Antibodies
☒	☐ Eukaryotic cell lines
☒	☐ Palaeontology and archaeology
☒	☐ Animals and other organisms
☒	☐ Clinical data
☒	☐ Dual use research of concern
☒	☐ Plants

Methods

n/a	Involved in the study
☒	☐ ChIP-seq
☒	☐ Flow cytometry
☒	☐ MRI-based neuroimaging

This checklist template is licensed under a Creative Commons Attribution 4.0 International License, which permits use, sharing, adaptation, distribution and reproduction in any medium or format, as long as you give appropriate credit to the original author(s) and the source, provide a link to the Creative Commons license, and indicate if changes were made. The images or other third party material in this article are included in the article's Creative Commons license, unless indicated otherwise in a credit line to the material. If material is not included in the article's Creative Commons license and your intended use is not permitted by statutory regulation or exceeds the permitted use, you will need to obtain permission directly from the copyright holder. To view a copy of this license, visit http://creativecommons.org/licenses/by/4.0/
